# Occupational neurotoxicant exposures, cognitive reserve, and brain aging: a scoping review

**DOI:** 10.3389/fpubh.2026.1971914

**Published:** 2026-09-29

**Authors:** Noah Leton, Sudel Bayley

**Affiliations:** Department of Physiology, Neuroscience and Behavioral Sciences, School of Medicine, St. George’s University, St. George's, Grenada

**Keywords:** brain aging, cognitive reserve, neuroimaging, neurotoxicity, occupational health, occupational neurotoxicant exposure

## Abstract

**Background:**

Occupational neurotoxicant exposure may contribute to cognitive decline, neurological dysfunction, and brain aging. However, the role of cognitive reserve in explaining differences in cognitive and neurological outcomes among exposed workers remains insufficiently characterized.

**Objective:**

This scoping review aimed to map evidence linking occupational neurotoxicant exposure with cognitive reserve or brain aging outcomes in adults, characterize exposure categories, cognitive reserve operationalizations, and neuroimaging methodologies, and identify evidence gaps.

**Methods:**

Searches were conducted in PubMed/MEDLINE, Scopus, Embase, and APA PsycInfo for English-language studies published from January 2011 onward. The review followed Joanna Briggs Institute guidance for scoping reviews and PRISMA-ScR reporting standards. Eligible studies examined adult populations with occupational neurotoxicant exposure and cognitive reserve or brain aging outcomes.

**Results:**

Sixteen studies met the inclusion criteria. Exposure categories included aluminum, lead, combustion-related exposures, organic solvents, and mixed-metal exposures. Cross-sectional and case–control designs predominated, and biological monitoring was the most frequently used exposure assessment approach. Cognitive decline or impairment was the most common brain aging outcome, while neuroimaging studies identified functional connectivity, white matter, cortical thickness, and brain volume alterations. Six studies did not explicitly operationalize cognitive reserve; among the remaining studies, approaches included educational attainment, biological susceptibility factors, and neuroimaging-based measures of brain structure or function. China was the most represented country, while evidence from other low- and middle-income regions remained limited.

**Conclusion:**

Diverse occupational neurotoxicant exposures were associated with adverse cognitive and neurological findings relevant to brain aging, although causality cannot be established. Cognitive reserve remains inconsistently examined, and neuroimaging measures should not be interpreted as direct measures of cognitive reserve. Future research should prioritize longitudinal studies integrating repeated exposure assessment, cognitive evaluation, multidimensional cognitive reserve measures, and multimodal neuroimaging.

## 1 Introduction

Cognitive aging and dementia represent major public health challenges of the twenty-first century. Globally, more than 55 million people are living with dementia, a figure projected to rise to 139 million by 2050 as populations age and life expectancy increases across both high-income and low- and middle-income countries (LMICs) (1, 2). Beyond dementia, cognitive decline, encompassing impairments in memory, executive function, and processing speed, affects a substantial proportion of the aging population and may precede clinically apparent neurodegenerative disease (3). As retirement ages increase and workforce participation extends into later decades of life, increasing attention has been directed toward identifying modifiable determinants of cognitive aging embedded within the occupational and environmental contexts of adult life (4–6).

Central to understanding individual variability in cognitive aging is the construct of cognitive reserve, broadly defined as the capacity to maintain cognitive functioning despite accumulating neuropathological burden or age-related brain changes (7, 8). Distinct from brain reserve, which refers primarily to the structural capacity of the brain to tolerate pathological insult, cognitive reserve reflects an active process through which the brain recruits and adapts cognitive processes and neural networks in response to aging or disease (9, 10). Cognitive reserve develops across the life course and is influenced by experiences such as education, occupational engagement, and other forms of cognitive enrichment (8, 11). Although the contribution of cognitively enriching experiences to cognitive reserve has been extensively studied, comparatively less attention has been directed toward factors that may influence differences in cognitive and neurological outcomes among individuals exposed to potentially neurotoxic environments over prolonged periods.

Occupational neurotoxicant exposures represent an important potential contributor to adverse neurological and cognitive outcomes. These exposures encompass diverse agents, including heavy metals such as lead, mercury, manganese, and arsenic; organic solvents such as benzene, toluene, and trichloroethylene; pesticides including organophosphates and pyrethroids; and combustion-related pollutants such as welding fumes and occupational particulate matter. Neurotoxicants may affect the central nervous system through mechanisms involving oxidative stress, neuroinflammation, mitochondrial dysfunction, and other pathways implicated in neuronal injury and neurodegeneration (12, 13). Occupational exposure to hazardous neurotoxic substances occurs across numerous industries, including agriculture, mining, manufacturing, and construction, with substantial exposure burdens reported in LMICs (4, 14). Epidemiological evidence has linked chronic occupational neurotoxicant exposure to adverse cognitive and neurological outcomes, including cognitive decline, mild cognitive impairment, and neurodegenerative disease, although the strength and nature of these associations vary according to the neurotoxicant, exposure characteristics, population, and outcome examined (15–18). Because occupational exposures may accumulate over prolonged working lives, neurological consequences may also emerge after substantial periods of exposure, including during later adulthood (19).

Despite the growing literature on occupational neurotoxicant exposure and cognitive and neurological outcomes, and the established literature on cognitive reserve, their intersection remains comparatively underexplored. Existing research has examined occupational and environmental neurotoxicants in relation to cognitive decline, dementia, and other neurological outcomes (15–18), while separate bodies of research have characterized education, occupational complexity, and related life-course factors as indicators or contributors to cognitive reserve (8, 11). Individual studies nevertheless suggest that reserve-related characteristics may influence the cognitive or neurological consequences of occupational neurotoxicant exposure. For example, Bleecker et al. (20) demonstrated that cognitive reserve moderated the dose-effect relationship between chronic occupational lead exposure and neurobehavioral performance among lead smelter workers, while Sabbath et al. (21) found that occupational solvent exposure was associated with cognitive impairment among workers with lower educational attainment but not among those with higher educational attainment. These findings suggest that individual differences in cognitive reserve or related characteristics may be relevant to understanding variation in neurological outcomes following occupational neurotoxicant exposure. However, the available evidence has not been systematically mapped across neurotoxicant exposure categories, cognitive reserve operationalizations, neuroimaging methodologies, and brain aging outcomes.

Given the emerging and methodologically heterogeneous literature linking occupational neurotoxicant exposure, cognitive reserve, and brain aging, a scoping review approach was considered appropriate for systematically mapping the available evidence and identifying important gaps in the literature. Accordingly, this review was guided by the methodological framework proposed by Arksey and O'Malley (22) and refined by the Joanna Briggs Institute (23), and was reported following the PRISMA extension for Scoping Reviews (PRISMA-ScR) checklist (24). The objectives of this review were: (1) to systematically map the existing evidence on associations between occupational neurotoxicant exposures and cognitive reserve or brain aging outcomes in adult populations; (2) to characterize the neurotoxicant exposure categories, cognitive reserve operationalizations, and neuroimaging methodologies represented in the identified literature; and (3) to identify key evidence gaps and inform priorities for future research on occupational neurotoxicant exposure, cognitive reserve, and brain aging. Specifically, this review sought to address the following research questions:

1. What is the current state of evidence on associations between occupational neurotoxicant exposures and cognitive reserve or brain aging outcomes in adult populations?
2. Which neurotoxicant exposure categories, cognitive reserve operationalizations, and neuroimaging methodologies are represented in this literature?
3. What evidence gaps are identified in the literature?

## 2 Methods

The objectives, inclusion criteria, and methodological procedures for this review were documented in a protocol registered on the Open Science Framework (OSF) during the conduct of the review (OSF registration: https://doi.org/10.17605/OSF.IO/ETHQ3). The review was conducted in accordance with JBI guidance for scoping reviews (23) and reported following the Preferred Reporting Items for Systematic Reviews and Meta-Analyses extension for Scoping Reviews (PRISMA-ScR) checklist [(24); Supplementary file 1].

### 2.1 Search strategy

A comprehensive literature search was conducted across four electronic bibliographic databases: PubMed/MEDLINE, Scopus, Embase, and APA PsycInfo. These databases were selected to ensure broad disciplinary coverage across occupational health, environmental toxicology, neuroscience, cognitive aging, and geriatric medicine. Web of Science Core Collection was not searched due to institutional access constraints. The search was restricted to studies published from January 2011 onward as a pragmatic eligibility decision to capture contemporary research informed by established cognitive reserve frameworks and modern occupational exposure assessment approaches. This cutoff does not imply that cognitive reserve frameworks originated during this period; rather, it reflects a decision to focus on evidence published following the broader uptake of these frameworks in occupational research. No upper publication date limit was applied. Only studies published in English were considered eligible for inclusion. To enhance comprehensiveness, the reference lists of all included studies were additionally screened to identify potentially relevant sources not retrieved through the primary database searches.

The search strategy incorporated free-text terms and controlled vocabulary relating to three principal concept areas: occupational neurotoxicant exposure; cognitive reserve and related reserve or resilience constructs; and brain aging outcomes. Search terms within each concept domain were combined using the Boolean operator OR, while the three concept domains were subsequently combined using the Boolean operator AND, with truncation symbols applied where appropriate to capture variant spellings and word endings. For PsycInfo, searched via the EBSCO platform, language and date restrictions were applied as platform limiters through the Search Options interface rather than embedded within the search string, consistent with EBSCO platform conventions. Complete search strategies for all databases are provided in Supplementary file 2. The cognitive reserve concept block was deliberately constructed to include broad neuroimaging and brain aging terms, including functional connectivity, cortical thickness, white matter integrity, neuroprotective, protective factors, and effect modification, that appear in studies examining brain structure or function without explicitly using the term cognitive reserve. Studies without explicit reserve measures were therefore retrievable through these broader terms within the cognitive reserve block.

### 2.2 Source screening and selection

All retrieved records were exported in RIS format and imported into Zotero reference management software (Corporation for Digital Scholarship) for organization and reference management. Records were subsequently uploaded to Rayyan systematic review software for automated duplicate detection and screening. Study selection was conducted in two stages. First, titles and abstracts were screened against the predefined inclusion and exclusion criteria by two independent reviewers (NL and SB). Full-text articles of studies considered potentially relevant were subsequently retrieved and assessed for eligibility. Any disagreements regarding study eligibility were resolved through discussion and consensus. The study selection process is summarized in the PRISMA-ScR flow diagram presented in Figure 1.

**Figure 1:**
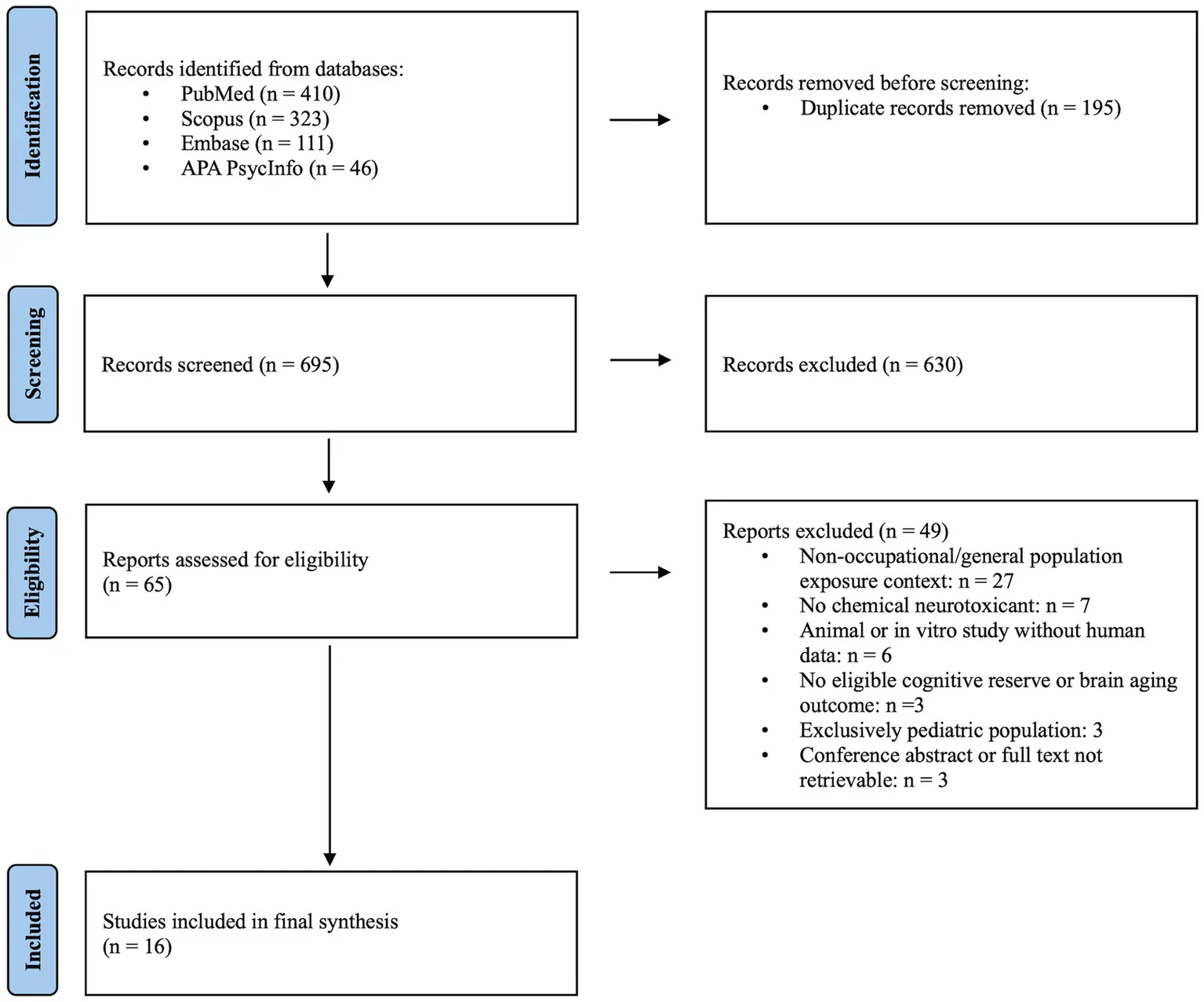
PRISMA–ScR flow diagram of study selection.

### 2.3 Inclusion and exclusion criteria

Eligibility criteria were developed according to the Population–Concept–Context (PCC) framework recommended by the Joanna Briggs Institute for scoping reviews (23). A summary of the inclusion and exclusion criteria is presented in Table 1.

**Table 1:** Inclusion and exclusion criteria based on the PCC framework.

Criteria	Inclusion	Exclusion
Population	Adult human participants aged 18 years or older with documented or self-reported occupational neurotoxicant exposure histories, regardless of current employment status; both active workers and retired individuals with prior occupational exposure histories; no restrictions applied based on sex, health status, or geographical location.	Animal studies; *in vitro* or laboratory studies not involving human participants; studies involving exclusively pediatric populations without adult follow-up data.
Concept—Exposure	Studies examining at least one occupational neurotoxicant exposure including heavy metals such as lead, mercury, manganese, arsenic, and cadmium; aluminum and aluminum compounds; organic solvents including benzene, toluene, styrene, and trichloroethylene; pesticides including organophosphates, carbamates, and pyrethroids; and combustion byproducts including occupational particulate matter, welding fumes, and polycyclic aromatic hydrocarbons.	Studies examining exclusively non-occupational environmental exposures without an occupational context or general population studies that did not document or assess occupational exposure histories; studies exclusively investigating occupational cognitive complexity as a reserve-building proxy without any chemical neurotoxicant exposure component.
Concept—Outcome	Studies reporting at least one measure of cognitive reserve including proxy-based measures such as educational attainment, premorbid intellectual ability, and occupational complexity; neuroimaging-based measures including functional connectivity, cortical thickness, white matter integrity, and brain age gap; composite reserve indices; or brain aging outcomes including cognitive decline, mild cognitive impairment, dementia and its subtypes, neurodegeneration, and related neuropsychological and neurobehavioral outcomes.	Studies reporting exclusively structural neuroimaging outcomes without any cognitive reserve or brain aging component; purely pharmacological or therapeutic intervention studies without occupational exposure assessment; studies reporting exclusively cardiovascular, metabolic, renal, or other non-neurological outcomes.
Concept—Review focus	All study designs including longitudinal cohort, cross-sectional, case–control, and review studies.	Conference abstracts, editorials, letters, commentaries, and opinion pieces.
Context	Any geographical setting including community, occupational, clinical, and epidemiological contexts	No exclusions based on setting or country of origin
Language	English language publications only.	Publications in languages other than English.
Date	Studies published from January 2011 onwards.	Studies published before January 2011.

#### 2.3.1 Population

Studies involving adult human participants aged 18 years or older with documented or self-reported occupational neurotoxicant exposure histories were considered eligible for inclusion, regardless of current employment status. Both active workers and retired individuals with prior occupational exposure histories were included. Studies examining both sexes were eligible, and sex was designated as a moderator variable for data extraction given established sex differences in neurotoxicant metabolism and cognitive reserve capacity. No restrictions were imposed with respect to health status or geographical location. Studies involving exclusively pediatric populations without adult follow-up data were excluded.

#### 2.3.2 Concept

Eligible studies examined associations between occupational neurotoxicant exposure and measures of cognitive reserve or brain aging outcomes. Relevant occupational neurotoxicant exposures included heavy metals such as lead, mercury, manganese, arsenic, and cadmium; aluminum and aluminum compounds; organic solvents including benzene, toluene, styrene, and trichloroethylene; pesticides such as organophosphates, carbamates, and pyrethroids; and combustion byproducts including occupational particulate matter, welding fumes, and polycyclic aromatic hydrocarbons. Eligible cognitive reserve operationalizations included proxy-based measures such as educational attainment, premorbid intellectual ability, and occupational complexity; neuroimaging-based measures including functional connectivity, cortical thickness, white matter integrity, and brain age gap; and composite reserve indices. For the purposes of this review, brain aging outcomes were defined broadly to encompass measurable cognitive, neuroimaging, neurobiological, and clinical indicators of age-related or pathological brain change. Eligible brain aging outcomes therefore included cognitive decline, mild cognitive impairment, dementia and its subtypes, neurodegeneration, structural and functional neuroimaging changes, neural damage biomarkers, and related neuropsychological and neurobehavioral outcomes. This broad definition was adopted to enable comprehensive mapping of the available evidence rather than to imply equivalence among these heterogeneous outcome measures. Studies reporting brain aging outcomes without an explicit cognitive reserve measure were considered eligible, as mapping the full evidence base, including studies that did not operationalize reserve, was necessary to identify gaps in how cognitive reserve has been examined across occupational neurotoxicant exposures. Studies exclusively investigating occupational cognitive complexity as a reserve-building proxy without any chemical neurotoxicant exposure component were excluded. Studies in which the primary outcome was not a cognitive reserve measure or brain aging outcome, including studies reporting exclusively cardiovascular, metabolic, renal, or other non-neurological outcomes, were also excluded.

#### 2.3.3 Context

Studies conducted across a range of geographical and research settings, including community, occupational, clinical, and epidemiological contexts, were eligible for inclusion. No restrictions were imposed based on country or study setting. Sources were excluded if they: (1) examined exclusively non-occupational environmental exposures without an occupational context, or were general population studies that did not document or assess occupational exposure histories; (2) were animal or *in vitro* studies without human participant data; (3) were published before January 2011; (4) were not published in English; or (5) consisted of conference abstracts, editorials, commentaries, letters, or opinion pieces.

### 2.4 Data extraction

Data were extracted from the included studies using a standardized data-charting form developed at the outset of data extraction and applied consistently across all studies. Data extraction was performed independently by two reviewers (NL and SB), with any discrepancies resolved through discussion and consensus. The charting form was refined during the extraction process to ensure consistency and relevance to the review objectives. Extracted data included author and publication details, study design and setting, participant characteristics including sample size, age range, sex distribution, and occupational group or industry; neurotoxicant exposure type and assessment method; cognitive reserve operationalization and specific measure or instrument used; brain aging outcome and neuroimaging modality where applicable; principal findings including direction and magnitude of association; moderating factors including sex, age, education, and cumulative exposure burden; and study limitations or research gaps identified by the authors.

### 2.5 Analysis and presentation of results

Findings from the included studies were synthesized using a descriptive analytical approach consistent with JBI guidance for scoping reviews (23), employing frequency-based quantitative mapping and descriptive qualitative categorization rather than meta-analysis or formal evidence synthesis. The evidence base was mapped according to seven key dimensions: neurotoxicant exposure category; cognitive reserve operationalization; study design; geographic location and occupational population; publication year; exposure assessment method; and brain aging outcome reported. Findings from small subgroups were reported descriptively rather than aggregated. Descriptive qualitative categorization employed structured coding to characterize each study’s operationalization of cognitive reserve, the mechanistic pathways proposed by study authors, and author-identified study limitations. Formal risk-of-bias assessment was not conducted, consistent with scoping review methodology. Results were organized and presented in alignment with the three review objectives and corresponding research questions, with narrative summaries supplemented by tabular presentations where appropriate. Where applicable, findings were additionally presented in relation to the Population–Concept–Context framework to facilitate interpretation across neurotoxicant exposure categories and reserve operationalization types. Emerging patterns, methodological gaps, and areas requiring further investigation were identified through narrative synthesis to inform future research directions in occupational neuroscience and cognitive aging.

## 3 Results

### 3.1 Search results

Database searches were conducted in July 2026 across four electronic bibliographic databases: PubMed/MEDLINE (*n* = 410), Scopus (*n* = 323), Embase (*n* = 111), and APA PsycInfo (*n* = 46). A total of 890 records were retrieved. Following importation into Zotero for reference management and subsequently into Rayyan for duplicate detection and screening, 195 duplicate records were identified and removed, yielding 695 unique records for title and abstract screening. All 695 records were independently screened by two reviewers (NL and SB) against the predefined inclusion and exclusion criteria. A total of 630 records were excluded at the title and abstract stage, and the remaining 65 reports were retrieved for full-text assessment. Of these, 49 were excluded following full-text review based on the predefined exclusion criteria outlined in Table 1. The primary reasons for exclusion were: non-occupational or general population exposure context (*n* = 27); no chemical neurotoxicant exposure (*n* = 7); animal or *in vitro* study without human participant data (*n* = 6); no eligible cognitive reserve or brain aging outcome (*n* = 3); exclusively pediatric population (*n* = 3); and conference abstract or full text not retrievable (*n* = 3). A total of 16 studies met all inclusion criteria and were included in the final synthesis. The study selection process is summarized in the PRISMA-ScR flow diagram presented in Figure 1.

### 3.2 Inclusion of sources of evidence

A total of 16 studies met the inclusion criteria and were included in the final synthesis. A summary of the characteristics of the included studies is presented in Table 2, with detailed study characteristics provided in Supplementary Table 1.

**Table 2:** Summary characteristics of included studies (*n* = 16).

Characteristic	Category	*n*	%
Year of publication	2011–2015	2	12.5
	2016–2020	5	31.3
	2021–2026	9	56.3
Country of origin	China	6	37.5
	USA	3	18.8
	South Korea	1	6.3
	France	1	6.3
	Mexico	1	6.3
	the Netherlands	1	6.3
	Italy	1	6.3
	USA/South Korea	1	6.3
	Multi-national	1	6.3
Study design	Cross-sectional	7	43.8
	Case–control	4	25.0
	Longitudinal cohort	2	12.5
	Prospective cohort	1	6.3
	Retrospective cohort	1	6.3
	Meta-analysis	1	6.3
Neurotoxicant exposure category	Aluminum	4	25.0
	Lead	3	18.8
	Organic solvents	2	12.5
	Combustion-related exposures	3	18.8
	Mixed metals	2	12.5
	Multiple exposures/other	2	12.5
Exposure assessment	Biological monitoring	9	56.3
	Air/environmental monitoring	1	6.3
	Self-reported occupational history	2	12.5
	Job-exposure matrix	2	12.5
	Other	2	12.5
Cognitive reserve operationalization	Neuroimaging-based	7	43.8
	Proxy-based—educational attainment	2	12.5
	Biological susceptibility	1	6.3
	None explicit	6	37.5
Brain aging outcome	Cognitive decline or impairment	11	68.8
	Brain structural change	3	18.8
	Other neurological outcome	2	12.5
Geographic setting	Low- and middle-income countries	7	43.8
	High-income countries	8	50.0
	Multi-national	1	6.3

Full study characteristics are presented in Supplementary Table 1. Country of origin refers to the country in which the study was conducted or the primary country of the study population. Studies described by the authors as cross-sectional case–control designs were classified as case–control studies. Income classifications follow World Bank criteria; multinational refers to the meta-analysis spanning multiple country income settings. Neurotoxicant exposure categories were assigned according to the primary or predominant exposure investigated. Studies involving multiple neurotoxicants or co-exposures were classified according to the predominant exposure. The “Multiple exposures/other” category includes studies examining multiple chemical exposures or ionizing radiation with concurrent chemical co-exposure. Other exposure assessment methods include bone lead measurement, multiple combined methods, and estimated flight hours. Biological susceptibility refers to genetic polymorphisms examined as potential modifiers of brain structural trajectories in James et al. (30), which may more accurately reflect biological susceptibility than cognitive reserve. Other neurological outcomes include neural damage biomarkers, and neurodegenerative mortality.

All included studies examined occupational exposure to one or more neurotoxicants and reported cognitive or neurological outcomes relevant to cognitive reserve, brain aging, or both. Collectively, these studies provide the evidence base examining associations between occupational neurotoxicant exposure, cognitive reserve, and brain aging across diverse occupational settings and populations.

### 3.3 Distribution of evidence

The distribution of included studies across key characteristics is summarized in Table 2. A summary evidence map of the included studies across key methodological and outcome dimensions is presented in Table 3.

**Table 3:** Evidence map of included studies (*n* = 16) across key methodological and outcome dimensions, grouped by neurotoxicant exposure category.

Study	Occupational population	Design	Exposure assessment	Cognitive reserve	Brain aging outcome	Neuroimaging	Reserve explicit
Aluminum (*n* = 4)							
(25)	Aluminum factory workers	Case–control	Biological monitoring	Neuroimaging-based	Cognitive decline/impairment	Yes	Partial
(26)	Aluminum factory workers	Case–control	Biological monitoring	Proxy—education	Cognitive decline/impairment	No	Yes
(27)	Aluminum factory workers	Cross-sectional	Biological monitoring	Neuroimaging-based	Cognitive decline/impairment	Yes	Partial
(28)	Aluminum factory workers	Cross-sectional	Biological monitoring	Neuroimaging-based	Cognitive decline/impairment	Yes	Partial
Lead (*n* = 3)							
(29)	Mixed occupational populations	Meta-analysis	Biological monitoring	None explicit	Cognitive decline/impairment	No	No
(30)	Former organolead manufacturing workers	Longitudinal cohort	Other	Biological susceptibility	Brain structural change	Yes	No
(31)	Lead smelting workers	Cross-sectional	Biological monitoring	Neuroimaging-based	Cognitive decline/impairment	Yes	Partial
Organic solvents (*n* = 2)							
(21)	GAZEL cohort—gas and electricity workers	Prospective cohort	Job-exposure matrix	Proxy—education	Cognitive decline/impairment	No	Yes
(35)	Paint factory workers	Cross-sectional	Air monitoring	None explicit	Cognitive decline/impairment	No	No
Combustion byproducts (*n* = 3)							
(32)	Firefighters	Longitudinal cohort	Biological monitoring	Neuroimaging-based	Brain structural change	Yes	Partial
(33)	Older adults with occupational exhaust fume exposure	Cross-sectional	Self-reported	None explicit	Cognitive decline/impairment	No	No
(34)	Aircrew	Case–control	Other	Neuroimaging-based	Cognitive decline/impairment	Yes	Partial
Mixed metals (*n* = 2)							
(36)	Steel mill workers	Cross-sectional	Biological monitoring	None explicit	Other neurological outcome	No	No
(37)	Mixed metal exposed workers—welding fumes	Cross-sectional	Biological monitoring	Neuroimaging-based	Brain structural change	Yes	Partial
Multiple chemical classes/other (*n* = 2)							
(38)	Early-onset dementia patients and controls	Case–control	Self-reported	None explicit	Cognitive decline/impairment	No	No
(39)	Uranium processing workers	Retrospective cohort	Job-exposure matrix	None explicit	Other neurological outcome	No	No

Neuroimaging column: Yes = neuroimaging used; No = not used. Reserve Explicit column: Yes = cognitive reserve explicitly operationalized; Partial = neuroimaging used as a reserve-related measure (not a direct measure of cognitive reserve); No = none explicit. Cognitive Reserve column: Neuroimaging-based = neuroimaging measures used as reserve-related indicators; Proxy—education = educational attainment used as reserve proxy; Biological susceptibility = genetic polymorphisms reflecting biological susceptibility rather than cognitive reserve; None explicit = cognitive reserve not operationalized. Full study characteristics are presented in Supplementary Table 1.

A total of 16 studies published between 2011 and 2026 were included in the final synthesis, with the majority published between 2021 and 2026 (*n* = 9, 56.3%), reflecting the recent and rapidly expanding nature of research in this field. The remaining studies were published between 2016 and 2020 (*n* = 5, 31.3%) and between 2011 and 2015 (*n* = 2, 12.5%).

Geographically, China was the most represented country of origin (*n* = 6, 37.5%), followed by the United States (*n* = 3, 18.8%). Single-country contributions were also identified from South Korea, France, Mexico, the Netherlands, and Italy (*n* = 1 each, 6.3%). One study was conducted across two countries—the United States and South Korea (*n* = 1, 6.3%)—and one multinational meta-analysis was identified (*n* = 1, 6.3%). More than two-fifths of the included studies originated from low- and middle-income countries (*n* = 7, 43.8%), predominantly China and Mexico, while half were conducted in high-income country settings (*n* = 8, 50.0%), with one multinational meta-analysis spanning multiple country income settings (*n* = 1, 6.3%).

Cross-sectional studies predominated, accounting for nearly half of all included studies (*n* = 7, 43.8%), followed by case–control studies (*n* = 4, 25.0%). Longitudinal cohort designs were represented by two studies (*n* = 2, 12.5%), with single contributions from prospective cohort, retrospective cohort, and meta-analytic designs (*n* = 1 each, 6.3%).

Aluminum was the most frequently investigated individual neurotoxicant, with four studies (*n* = 4, 25.0%) focusing specifically on occupational aluminum exposure, all conducted among aluminum factory workers in China. An additional study examined aluminum alongside other chemical exposures. Lead was examined in three studies (*n* = 3, 18.8%), including workers in organolead manufacturing, smelting, and a multinational meta-analysis. Combustion-related and other occupational chemical exposures were examined in three studies (*n* = 3, 18.8%), including polycyclic aromatic hydrocarbons among firefighters, occupational exhaust fumes, and organophosphate exposure associated with aircraft engine oil. Organic solvents and mixed-metal exposures were each investigated in two studies (*n* = 2, 12.5%). Two additional studies examined broader exposure groupings: one involved multiple chemical exposures, while the other investigated ionizing radiation with concurrent chemical co-exposure (*n* = 2, 12.5%).

Biological monitoring was the most frequently employed exposure assessment approach (*n* = 9, 56.3%), comprising measurements of plasma aluminum, blood lead, whole blood metals and urinary metals or polycyclic aromatic hydrocarbon metabolites. Job-exposure matrices and self-reported occupational exposure histories were each used in two studies (*n* = 2, 12.5%), while personal air sampling for organic solvents was employed in one study (*n* = 1, 6.3%). The remaining studies used alternative approaches, including bone lead measurements and estimated flight hours (*n* = 2, 12.5%).

With respect to cognitive reserve and reserve-related measures, neuroimaging-based approaches were the most frequently represented (*n* = 7, 43.8%), including measures of functional connectivity, white matter integrity, cortical thickness, and diffusion tensor imaging metrics. Six studies (*n* = 6, 37.5%) did not explicitly operationalize cognitive reserve. Two studies used educational attainment as a proxy measure of cognitive reserve (*n* = 2, 12.5%), while one study reported biological susceptibility factors (*n* = 1, 6.3%).

Brain aging outcomes were most commonly assessed through measures of cognitive decline or impairment (*n* = 11, 68.8%), including validated cognitive screening instruments such as the Montreal Cognitive Assessment, the Digit Symbol Substitution Test, and comprehensive neuropsychological and sensorimotor test batteries, as well as clinically diagnosed early-onset dementia and cognitive impairment assessed alongside neuroimaging findingsStructural brain changes, including reductions in cortical thickness, brain volume, and alterations in medial temporal lobe diffusion metrics, were reported in three studies (*n* = 3, 18.8%). Two studies (*n* = 2, 12.5%) reported other neurological outcomes, including biomarkers of neural injury and neurodegenerative mortality.

### 3.4 Research question 1: current state of evidence on occupational neurotoxicant exposures and cognitive reserve or brain aging outcomes in adult populations

The 16 included studies collectively reported associations between occupational neurotoxicant exposure and adverse cognitive or neurological outcomes across a range of occupational populations. Although the neurotoxicants examined, exposure assessment methods, and outcome measures varied, the included studies consistently evaluated cognitive performance, structural or functional brain changes, or both in relation to occupational exposure.

Five studies included occupational aluminum exposure, of which four focused specifically on aluminum exposure, and one examined aluminum alongside other chemical exposures. Zhao et al. (25) reported that reduced default mode network functional connectivity partially mediated the association between plasma aluminum concentration and cognitive performance measured using the Montreal Cognitive Assessment (MoCA). Meng et al. (26) found that higher plasma aluminum concentrations were associated with an increased risk of mild cognitive impairment, while higher educational attainment was associated with lower odds of impairment. Two subsequent studies by Zhang et al. (27, 28) reported that occupational aluminum exposure was associated with lower MoCA scores, altered functional connectivity within executive control and limbic networks, and changes in white matter integrity involving the fornix and stria terminalis.

Evidence relating to occupational lead exposure was reported in three studies. Vlasak et al. (29) synthesized evidence from 22 studies and reported an association between higher blood lead concentrations and poorer neurocognitive and sensorimotor performance. James et al. (30) examined former organolead workers and reported associations between selected genetic polymorphisms and differences in white matter volume decline and progression of white matter lesions. Shi et al. (31) reported that blood lead concentrations among smelting workers were associated with altered insular functional connectivity together with lower cognitive screening scores measured using the MoCA and Mini-Mental State Examination.

Three studies examined combustion-related occupational exposures. Kim et al. (32) reported that urinary polycyclic aromatic hydrocarbon metabolites among firefighters were associated with reductions in cortical thickness and subcortical brain volume over 2 years. Liang et al. (33) reported that occupational exhaust fume exposure was associated with lower performance across multiple cognitive domains, including delayed memory, verbal fluency, and processing speed. Reneman et al. (34) examined aircrew members exposed to organophosphates from aircraft engine oil and reported cognitive impairment along with alterations in white matter microstructure.

Studies examining organic solvents also reported associations with cognitive outcomes. Juárez-Pérez et al. (35) found that cumulative exposure to toluene, xylene, and benzene among paint factory workers was associated with poorer neuropsychological and sensorimotor performance. Similarly, Sabbath et al. (21) reported that occupational solvent exposure was associated with cognitive impairment among workers with lower educational attainment, whereas this association was not observed among workers with higher educational attainment.

Mixed metal exposures were examined in two studies. Wu et al. (36) reported associations between multiple metal exposures and biomarkers of neural injury among steel mill workers, while Lee et al. (37) found that exposure to manganese- and copper-containing welding fumes was associated with alterations in medial temporal lobe diffusion measures and poorer neuropsychological performance.

The remaining evidence addressed broader occupational exposure groupings. Adani et al. (38) reported associations between occupational exposure to aluminum, pesticides, dyes, paints, and thinners and increased risk of frontotemporal dementia, whereas Zbysinski et al. (39) examined uranium processing workers and reported that machining fluid exposure modified the association between occupational radiation exposure and neurodegenerative mortality.

Collectively, the included studies reported associations between a range of occupational neurotoxicant exposure categories and brain aging outcomes, including cognitive decline, mild cognitive impairment, structural and functional neuroimaging changes, neural injury biomarkers, dementia, and neurodegenerative mortality. Evidence was identified across diverse occupational settings, including manufacturing, mining, welding, firefighting, aviation, and industrial processing.

### 3.5 Research question 2: neurotoxicant exposure categories, cognitive reserve operationalizations and neuroimaging methodologies

#### 3.5.1 Neurotoxicant exposure categories examined

The included studies represented a range of neurotoxicant exposure categories. Aluminum was the most frequently investigated individual neurotoxicant, with four studies focusing specifically on occupational aluminum exposure. An additional study examined aluminum alongside other chemical exposures. Lead was examined in three studies, while two studies investigated mixed-metal exposures involving manganese- and copper-containing welding fumes and multiple metal mixtures encountered among steel mill workers. Combustion-related exposures were represented in three studies, including polycyclic aromatic hydrocarbons among firefighters, occupational exhaust fumes, and aircraft engine oil fumes with potential organophosphate contamination. Organic solvents were examined in two studies. Two studies involved broader exposure groupings: Adani et al. (38) examined occupational exposure to aluminum, pesticides, dyes, paints, and thinners in relation to early-onset dementia, while Zbysinski et al. (39) investigated occupational ionizing radiation together with machining fluid co-exposure. Because some studies involved more than one neurotoxicant, these exposure categories were not mutually exclusive at the level of individual neurotoxicants.

Exposure assessment methodologies varied across the included studies, consistent with the diversity of occupational neurotoxicant exposures represented in the literature. Biological monitoring was the predominant exposure assessment approach and included plasma aluminum, blood lead, urinary metals, and urinary polycyclic aromatic hydrocarbon metabolite measurements. Additional methods included personal air monitoring, bone lead measurement, job-exposure matrices, self-reported occupational exposure histories, cumulative radiation dose estimation, and estimated flight hours. Juárez-Pérez et al. (35) was the only study to employ personal air monitoring to quantify occupational solvent exposure, while Zbysinski et al. (39) uniquely estimated cumulative occupational radiation dose in conjunction with machining fluid co-exposures.

#### 3.5.2 Cognitive reserve operationalizations

Neuroimaging-based approaches were the most frequently represented among studies incorporating cognitive reserve or reserve-related measures, appearing in seven of the included studies. These approaches included functional connectivity, cortical thickness, white matter integrity, diffusion tensor imaging metrics, and other structural and functional magnetic resonance imaging measures. However, these measures primarily characterized brain structure or function and were not necessarily direct measures of cognitive reserve. Educational attainment was used as a proxy for cognitive reserve in two studies. Meng et al. (26) and Sabbath et al. (21) both incorporated educational attainment to examine cognitive reserve in relation to occupational neurotoxicant exposure. James et al. (30) was the only study to investigate genetic polymorphisms, including APOE, ACE, and VDR, as potential modifiers of longitudinal brain structural changes among former organolead workers. These polymorphisms are classified as biological susceptibility factors rather than cognitive reserve measures, as genetic characteristics of this kind may more accurately reflect biological susceptibility than cognitive reserve as conceptualized by Stern et al. (8). Six studies did not explicitly operationalize cognitive reserve, instead reporting associations between occupational neurotoxicant exposure and cognitive or neurological outcomes.

#### 3.5.3 Neuroimaging methodologies

Magnetic resonance imaging (MRI) was the predominant neuroimaging methodology across the included studies and included structural MRI, functional MRI (fMRI), and diffusion tensor imaging (DTI). Functional MRI techniques, including rs-fMRI, were the most frequently employed neuroimaging approaches, appearing in four studies, and were primarily used to evaluate alterations in functional brain connectivity associated with occupational aluminum and lead exposure. Diffusion tensor imaging was employed in three studies to evaluate white matter integrity and medial temporal lobe microstructure, while structural MRI was used to assess cortical thickness, brain volume, and white matter lesion progression.

Neuroimaging methodologies varied according to the study objectives and neurotoxicants investigated. Kim et al. (32) employed longitudinal structural MRI to evaluate changes in cortical thickness and subcortical brain volume over a two-year follow-up period among firefighters. Zhang et al. (28) combined rs-fMRI and DTI to evaluate alterations in functional connectivity and white matter integrity among aluminum-exposed workers. Lee et al. (37) used DTI to examine medial temporal lobe diffusion characteristics associated with welding fume exposure.

### 3.6 Research question 3: evidence gaps in the current literature

Several important evidence gaps were identified across the included studies. First, although cognitive reserve was the central construct underpinning this review, six of the 16 included studies did not explicitly operationalize cognitive reserve despite examining occupational neurotoxicant exposure in relation to cognitive or neurological outcomes. These studies were retained because their findings were considered relevant to understanding the broader evidence base and to identifying gaps in cognitive reserve operationalization across occupational neurotoxicant exposure categories. Among the remaining studies, approaches to examining cognitive reserve and reserve-related characteristics varied. Educational attainment was represented in two studies, biological susceptibility factors in one study, and neuroimaging-based measures of brain structure or function in seven studies.

Second, only a small number of longitudinal studies were identified, with most of the evidence derived from cross-sectional and case–control study designs. Third, although the included studies represented a range of occupational neurotoxicant exposure categories, relatively few studies were available within each category. Aluminum was examined in four studies, lead and combustion-related exposures in three studies each, and organic solvents and mixed-metal exposures in two studies each. Notably, several eligible neurotoxicant classes were absent or underrepresented as primary exposures in the included literature, including mercury, cadmium, styrene, trichloroethylene, and pesticides. Furthermore, single timepoint biological monitoring was used in nine of the included studies, only two studies examined neurotoxicant mixtures or co-exposures, and no study systematically investigated cerebrovascular mechanisms.

Finally, the geographical distribution of the evidence was concentrated in China and high-income countries, with comparatively limited representation from low- and middle-income countries outside China. Collectively, these findings identify important evidence gaps in the operationalization of cognitive reserve, longitudinal investigation, occupational neurotoxicant representation, and geographical coverage of the current literature. Key evidence gaps and future research priorities identified in this review are summarized in Table 4.

**Table 4:** Key evidence gaps and research priorities for understanding occupational neurotoxicant exposure, cognitive reserve, and brain aging.

Priority area	Rationale	Recommended approach	Current evidence base
Longitudinal study designs	Cross-sectional and case–control designs predominated, limiting causal inference and the ability to characterize cognitive trajectories over time.	Prospective occupational cohort studies with repeated cognitive assessment, neuroimaging, and biological monitoring across the working life.	2 of 16 studies used longitudinal cohort designs; 7 used cross-sectional designs and 4 used case–control designs.
Standardized cognitive reserve operationalization	Approaches to cognitive reserve varied substantially across studies, limiting comparability and the ability to assess reserve as a modifier of neurotoxicant effects.	Adopt standardized multidimensional reserve measures combining proxy-based, neuroimaging-based, and composite indices across occupational neurotoxicant studies.	6 of 16 studies had no explicit reserve measure; 2 used proxy-based measures; 7 used neuroimaging-based measures; 1 reported biological susceptibility factors.
Repeated biological monitoring	Single timepoint biological monitoring may not accurately reflect cumulative or historical exposure levels, potentially underestimating the neurological consequences of prolonged occupational exposure.	Implement repeated biological monitoring across the working life to characterize cumulative exposure burden and its relationship with cognitive and neurological outcomes.	9 of 16 studies used single timepoint biological monitoring.
Mixed and co-exposure assessment	Occupational workers are frequently exposed to multiple neurotoxicants simultaneously, making it difficult to attribute neurological outcomes to a single agent when studied in isolation.	Apply mixture analysis methods such as Bayesian kernel machine regression and weighted quantile sum regression to assess joint neurotoxicant effects.	Only 2 of 16 studies examined mixed metal exposures; no study applied mixture analysis methods.
Geographic and occupational diversity	Concentration of evidence in China and high-income countries limits generalizability to diverse occupational populations and geographic settings with differing exposure profiles and regulatory protections.	Expand research to underrepresented regions including sub-Saharan Africa, South Asia, and Latin America, and to underrepresented occupational sectors.	6 of 16 studies from China; only 1 of 16 from an LMIC outside China.
Neuroimaging-based reserve measures	Neuroimaging measures reflect brain structure or function and should not be interpreted as direct measures of cognitive reserve without explicit conceptual framing.	Develop standardized neuroimaging protocols explicitly framed within cognitive reserve theory for use in occupational neurotoxicology research.	7 of 16 studies used neuroimaging as a reserve-related measure; none explicitly defined neuroimaging as a direct reserve measure.
Vascular mechanisms	Cerebrovascular pathways may link occupational exposures to age-related neurological outcomes but were not systematically examined in the included literature.	Incorporate cerebrovascular measures including cerebral small vessel disease markers and vascular risk factor assessment in future occupational neurotoxicology studies.	No included study systematically examined cerebrovascular mechanisms.
Underrepresented neurotoxicant classes	Several neurotoxicant classes listed as eligible exposures were absent or underrepresented as primary exposures, leaving important gaps in the occupational neurotoxicology evidence base.	Prioritize studies of occupational mercury, cadmium, styrene, trichloroethylene, and pesticide exposures in relation to cognitive reserve and brain aging outcomes.	No included study examined mercury, cadmium, styrene, trichloroethylene, or pesticides as a primary exposure.

Priorities are informed by evidence gaps identified across the 16 included studies. LMIC, low- and middle-income countries.

## 4 Discussion

This scoping review synthesized the available evidence on the relationship between occupational neurotoxicant exposure, cognitive reserve, and brain aging outcomes in adult populations. Across the 16 included studies, occupational exposure to metals, combustion-related pollutants, organic solvents, and mixed occupational exposures was associated with a range of adverse cognitive and neurological outcomes relevant to brain aging. The evidence base was heterogeneous with respect to study design, exposure assessment, cognitive reserve operationalization, neuroimaging methodology, and outcome measures. Nevertheless, the findings indicate a recurring pattern of cognitive impairment, structural and functional brain alterations, and other neurological indicators among occupationally exposed populations. These findings are broadly consistent with the wider literature linking neurotoxicant exposure with adverse cognitive and neurological outcomes, including cognitive decline and neurodegenerative disease (15–18, 40).

A particularly important finding was the limited integration of cognitive reserve within the literature included in this review. More than one-third of the included studies did not explicitly operationalize cognitive reserve, while studies that incorporated reserve or reserve-related measures used heterogeneous approaches, including educational attainment, biological susceptibility factors, and neuroimaging-based measures of brain structure or function. This limited and varied application of the cognitive reserve construct is notable given its established role in explaining interindividual differences in cognitive functioning in the presence of age-related or pathological brain changes (7, 8, 10). The findings therefore suggest that the literature has begun to incorporate concepts relevant to cognitive reserve but has not yet developed a consistent framework for examining how reserve may modify the neurological consequences of occupational neurotoxicant exposure.

Several important uncertainties emerge from these findings. First, although occupational neurotoxicant exposure was generally associated with adverse cognitive or neurological outcomes, the extent to which cognitive reserve modifies these associations remains incompletely understood. Second, the convergence of neurological outcomes across chemically diverse exposures raises the possibility of shared biological pathways, but the available evidence is insufficient to determine whether these pathways are common across neurotoxicants or partly exposure-specific. Third, although neuroimaging studies identified structural and functional brain alterations, the temporal relationship between these changes and subsequent cognitive decline remains uncertain. These uncertainties highlight the need for longitudinal and mechanistically informed research examining how occupational neurotoxicant exposure contributes to brain aging and how individual differences in cognitive reserve or other susceptibility factors may influence these trajectories.

### 4.1 Occupational neurotoxicant exposure and brain aging

The evidence synthesized in this review supports the growing recognition that occupational neurotoxicant exposure may represent an important environmental contributor to brain aging. Across diverse occupational settings, exposure to metals, combustion-related pollutants, organic solvents, and mixed neurotoxicants was associated with adverse cognitive and neurological outcomes, although the specific outcomes varied across studies. These included cognitive impairment, structural brain changes, functional connectivity alterations, and biomarkers of neural injury. The observation of neurological effects across different occupational neurotoxicant exposure categories raises the possibility that occupational exposure may contribute to brain aging through multiple, potentially overlapping pathways rather than through a single exposure-specific mechanism. Establishing whether occupational neurotoxicant exposure accelerates brain aging requires longitudinal evidence of change over time, which the predominantly cross-sectional and case–control designs in this review cannot provide.

An important observation was the convergence of neurological findings across chemically diverse occupational exposures. The neurotoxicants represented in the included studies differed substantially in their chemical properties, toxicokinetics, exposure pathways, and occupational contexts. Nevertheless, several were associated with cognitive dysfunction accompanied by structural, functional, or biological indicators of neurological injury. This pattern raises the possibility that occupational neurotoxicants may converge on common processes involved in neural injury and brain aging rather than giving rise to entirely distinct effects. Evidence from the broader neurotoxicology literature provides biological plausibility for overlapping mechanisms involving oxidative stress, neuroinflammation, mitochondrial dysfunction, and neuronal injury (12, 13, 41). It should be noted that these mechanistic pathways were not directly measured by the included studies and are drawn from the broader neurotoxicology literature to provide biological plausibility for the observed associations rather than to claim mechanistic evidence from the reviewed studies. However, because the included studies did not directly compare these mechanisms across neurotoxicant exposure categories, the extent to which such pathways account for the observed convergence remains uncertain. Cerebrovascular mechanisms represent an additional pathway warranting consideration. Vascular dysfunction and cerebral small vessel disease have been associated with cognitive decline and dementia in the broader literature, and some occupational exposures represented in this review, including lead and combustion-related pollutants, have been linked to cerebrovascular effects elsewhere (42). Explicit investigation of vascular pathways in future occupational neurotoxicology research would therefore strengthen understanding of how occupational exposures may contribute to brain aging.

The findings also indicate that the current literature primarily captures intermediate indicators of brain aging rather than clinically established neurodegenerative disease. Cognitive impairment, structural brain alterations, functional connectivity changes, and biomarkers of neural injury were more commonly reported than clinically diagnosed dementia or other neurodegenerative disorders. This pattern is consistent with contemporary models of brain aging in which biological and functional changes may precede overt clinical disease (3, 8). Accordingly, occupational neurotoxicant exposure may contribute to trajectories of brain aging-related change without necessarily manifesting as clinically apparent disease during the period in which exposure is assessed. From an occupational health perspective, this underscores the potential value of identifying early indicators of neurological injury and strengthening exposure prevention before clinically significant cognitive impairment develops. It also provides an important context for examining whether differences in cognitive reserve or other susceptibility factors influence how exposure-related neurological changes are expressed cognitively.

### 4.2 Cognitive reserve and differential vulnerability to occupational neurotoxicity

An important finding emerging from this review is that cognitive reserve provides a useful conceptual framework for understanding potential differences in cognitive and neurological outcomes among similarly exposed workers. The available evidence does not establish cognitive reserve as a demonstrated protective factor across occupational neurotoxicant exposures; rather, it suggests that reserve-related characteristics may help explain variation in susceptibility and cognitive expression. This distinction is important because relatively few included studies explicitly examined cognitive reserve as a modifying factor. Nevertheless, differences in outcomes among exposed populations are consistent with cognitive reserve theory, which proposes that lifetime cognitive enrichment influences the capacity to maintain cognitive function despite age-related or pathological brain changes (7, 8, 10).

The operationalizations identified in this review also demonstrate substantial variation in how reserve-related characteristics have been examined. Educational attainment was used as a proxy in studies such as Meng et al. (26) and Sabbath et al. (21), while neuroimaging-based measures of brain structure and function were more frequently represented across the included literature. James et al. (30) additionally examined genetic polymorphisms in relation to longitudinal changes in brain structure. These approaches capture different aspects of individual susceptibility and neural adaptation, but they should not be considered interchangeable measures of cognitive reserve. Educational attainment represents a relatively distal life-course proxy, whereas neuroimaging measures provide information about brain structure or function, and genetic characteristics may reflect biological susceptibility rather than reserve itself. Contemporary cognitive reserve frameworks similarly emphasize the distinction between reserve, brain reserve, and brain maintenance (8).

This distinction is particularly important when interpreting the neuroimaging studies included in this review. Neuroimaging abnormalities may indicate neural injury associated with occupational exposure, but they should not automatically be interpreted as evidence of reduced or preserved cognitive reserve. Rather, neuroimaging may provide information about the neural substrates through which reserve is expressed or through which exposure-related pathology becomes evident. For example, findings involving functional connectivity, white matter integrity, cortical thickness, and brain volume may help characterize the neural consequences of exposure, while their relationship to cognitive reserve requires integration with cognitive performance and established reserve-related characteristics.

Taken together, the findings support greater integration of cognitive reserve into occupational neurotoxicology research, while also highlighting the need for conceptual and methodological clarity. Future studies should distinguish cognitive reserve from related constructs and examine reserve using multidimensional approaches that combine appropriate life-course indicators with cognitive and neurobiological measures. Such approaches may help determine whether cognitive reserve modifies exposure–outcome relationships, identifies populations with greater or lesser susceptibility, or simply reflects broader differences in brain health. Importantly, the current evidence is insufficient to justify differential occupational protection based on cognitive reserve, educational attainment, or genetic characteristics. Their value at this stage is primarily in advancing understanding of why neurological outcomes may vary among workers with similar exposure histories.

### 4.3 Neuroimaging methodologies

The neuroimaging findings identified in this review provide evidence that occupational neurotoxicant exposure may be associated with alterations in both brain function and structure. Functional MRI was the most frequently represented neuroimaging approach. Studies of occupational aluminum exposure reported alterations in connectivity involving the default mode, executive-control, and limbic networks, while occupational lead exposure was associated with altered insular functional connectivity (25, 27, 31). These findings suggest that occupational neurotoxicant exposure may affect the organization and coordination of distributed neural networks involved in cognitive processing. Such alterations are relevant to brain aging because changes in large-scale functional organization have been associated with age-related cognitive decline (43, 44). Particularly informative was the finding by Zhao et al. (25) that altered default mode network connectivity partially mediated the association between aluminum exposure and cognitive performance, providing preliminary evidence of a potential neural pathway linking occupational exposure with cognitive outcomes.

Diffusion tensor imaging and structural MRI provided complementary evidence of microstructural and macroscopic brain alterations. Higher medial temporal diffusivity was associated with poorer neuropsychological performance among individuals with mixed-metal exposure, with parahippocampal fractional anisotropy contributing to the relationship between metal exposure and delayed story recall (37). Aluminum exposure was also associated with differences in white matter integrity involving the fornix/stria terminalis (28). In addition, occupational exposure was associated with cortical thickness and subcortical volume changes among firefighters and with longitudinal brain-volume decline and white matter lesion progression among former organolead workers (30, 32). Collectively, these findings suggest that occupational neurotoxicant exposure may be associated with alterations across multiple levels of neural organization, from functional connectivity and white matter microstructure to macroscopic brain morphology.

The convergence of functional, microstructural, and structural findings is particularly relevant to understanding occupational contributions to brain aging. However, these neuroimaging measures should primarily be interpreted as indicators of brain structure or function rather than as direct measures of cognitive reserve. Furthermore, the available evidence does not establish whether functional alterations precede structural injury, arise as compensatory responses, or represent consequences of more established neurological damage. Similarly, structural abnormalities may reflect cumulative occupational exposure effects but cannot, in the available studies, be definitively attributed to occupational neurotoxicant exposure alone. Repeated neuroimaging alongside longitudinal exposure assessment is therefore needed to clarify the temporal sequence of these changes.

Future research incorporating explicit cognitive reserve measures alongside multimodal neuroimaging and longitudinal exposure assessment would help clarify whether neural alterations represent early markers of injury, mechanisms linking exposure to cognitive impairment, or manifestations of differences in susceptibility.

### 4.4 Methodological challenges and future research directions

Several methodological challenges identified in this review constrain interpretation of the current evidence and define important priorities for future research. The most prominent is the predominance of observational designs, particularly cross-sectional and case–control studies. Seven of the 16 included studies were cross-sectional, while four were case–control studies. Four studies used cohort designs, including two classified as longitudinal cohort studies, one prospective cohort study, and one retrospective cohort study. Although studies such as Kim et al. (32) and James et al. (30) provide evidence of changes in brain structure over time, the limited longitudinal evidence makes it difficult to determine whether observed cognitive and neuroimaging abnormalities represent early effects of occupational exposure, cumulative consequences of prolonged exposure, or differences in pre-existing susceptibility. Prospective occupational cohorts with repeated exposure assessment, cognitive evaluation, and neuroimaging are therefore needed to establish temporal trajectories and identify potential early markers of neurotoxicant-related brain aging. The importance of longitudinal designs is further underscored by existing evidence linking cumulative lead exposure to longitudinal cognitive decline in older adults (45, 46), illustrating the value of prospective designs with repeated exposure assessment for characterizing cognitive trajectories associated with occupational neurotoxicant exposure.

A second challenge is substantial heterogeneity in exposure assessment, cognitive reserve operationalization, and neuroimaging methodology. Biological monitoring was the most frequently used exposure assessment approach, but job-exposure matrices, self-reported exposure histories, personal air sampling, bone lead measurements, and other approaches were also represented. A notable limitation of biological monitoring in this context is that most included studies obtained measurements at a single time point, which may not accurately reflect cumulative or historical exposure levels. Repeated biological monitoring would provide a more accurate characterization of exposure burden and its relationship with cognitive and neurological outcomes over time. Reserve and reserve-related characteristics were similarly assessed using heterogeneous approaches, including neuroimaging measures, educational attainment, and biological susceptibility factors, while six studies did not explicitly operationalize cognitive reserve. This variation limits direct comparison across studies and makes it difficult to determine whether differences in findings reflect true variation in neurotoxicant effects or differences in measurement approaches. Future studies would benefit from more consistent exposure characterization and clearly defined multidimensional approaches to cognitive reserve while retaining sufficient flexibility to investigate neurotoxicant-specific effects.

A third gap concerns the limited depth of evidence for individual neurotoxicant exposure categories. The included studies encompassed a range of occupational neurotoxicant exposure categories, with relatively few studies available within each category. Aluminum was examined in four studies, lead and combustion-related exposures in three studies each, and organic solvents and mixed-metal exposures in two studies each. This distribution limits the ability to determine whether observed associations are consistent within specific neurotoxicants or instead reflect effects shared across chemically diverse exposures. Notably, several neurotoxicant classes listed as eligible exposures, including mercury, cadmium, styrene, trichloroethylene, and pesticides, were absent or underrepresented as primary exposures in the included literature, representing important priorities for future occupational neurotoxicology research. Larger occupational cohorts with sufficient exposure-specific sample sizes are needed to examine dose–response relationships, exposure duration, latency, and potential differences in neurological effects across neurotoxicant exposure categories. Such studies should also apply established mixture analysis methods to account for co-exposures and assess the joint effects of multiple neurotoxicant exposures, more closely reflecting real-world occupational environments.

Finally, the geographical distribution of the evidence limits the generalizability of current findings. China was the most represented country, accounting for six of the 16 included studies. Additional studies were conducted in high-income settings, while representation of low- and middle-income countries outside China and Mexico remained limited. Occupational exposure profiles, regulatory protections, workplace practices, socioeconomic conditions, and access to health care may differ substantially across settings, potentially influencing both exposure intensity and neurological outcomes. Expanding research into underrepresented regions and occupational populations is therefore important for determining whether the relationships identified in the current literature are broadly applicable or context-dependent.

Taken together, these gaps indicate a need to move beyond predominantly isolated exposure–outcome associations toward longitudinal, multidimensional investigations. Future studies integrating quantitative exposure assessment, repeated cognitive assessment, multidimensional cognitive reserve measures, and multimodal neuroimaging could clarify not only whether occupational neurotoxicant exposure is associated with brain aging, but also when neural changes emerge, how individual susceptibility influences their cognitive expression, and which characteristics may identify workers at greater risk of adverse neurological consequences.

### 4.5 Implications for occupational health practice and policy

The findings of this review have important implications for occupational health practice and policy, particularly given the potential for occupational neurotoxicant exposure to contribute to cognitive and neurological changes over prolonged working lives. Although the evidence does not establish causality or support specific clinical interventions, the recurrence of adverse cognitive, structural, functional, and biomarker findings across several neurotoxicant exposure categories supports continued emphasis on primary prevention and exposure reduction. Occupational health strategies should therefore prioritize identification and control of neurotoxicant hazards through exposure monitoring, elimination or substitution where feasible, engineering and process controls, and appropriate use of personal protective equipment. These approaches are consistent with international occupational health guidance emphasizing prevention and control of hazardous chemical exposures (47–49).

The findings also highlight the potential value of occupational health surveillance among workers with sustained or cumulative exposure to recognized neurotoxicants (50). Periodic assessment of cognitive or neurological function may help identify emerging concerns in appropriately selected high-exposure populations; however, the current evidence is insufficient to recommend routine neuroimaging as a clinical screening tool. The fMRI, DTI, and structural MRI findings identified in this review should currently be regarded primarily as research measures that may help characterize subclinical neurological changes and clarify pathways linking occupational exposure with cognitive outcomes. Further longitudinal evidence is needed before these measures can be considered for integration into validated occupational surveillance or clinical decision-making tools.

At the policy level, the findings reinforce the importance of maintaining effective occupational exposure standards, exposure monitoring, worker training, and access to occupational health services. These measures are particularly important for neurotoxicants with potential cumulative effects, for which prevention of exposure is an important strategy for reducing the potential long-term neurological consequences of occupational exposure. International guidance emphasizes systematic assessment and monitoring of hazardous chemical exposures, worker information and training, and implementation of appropriate preventive measures (48).

Finally, the limited evidence concerning cognitive reserve and individual susceptibility suggests that workers with similar occupational exposure histories may experience different neurological or cognitive outcomes. However, the current evidence does not support differential occupational exposure standards based on cognitive reserve, educational attainment, or genetic characteristics. Rather, these findings underscore the importance of broad preventive protections for exposed workers while future research investigates factors that may modify vulnerability. Strengthening exposure prevention, longitudinal occupational health surveillance, and research capacity therefore represents an important priority in light of the emerging evidence linking occupational neurotoxicant exposure with cognitive and brain aging outcomes.

### 4.6 Review limitations

This scoping review has several limitations that should be considered when interpreting its findings. First, although a comprehensive search was conducted across four major bibliographic databases, relevant studies indexed in other databases may not have been identified.

Second, consistent with the scoping review methodology, no formal risk-of-bias or quality appraisal of individual studies was undertaken. Consequently, the findings should be interpreted as a mapping and synthesis of the available evidence rather than as an assessment of the strength or certainty of individual associations.

Third, the relatively small number of included studies limits the breadth of the available evidence. Although 16 studies met the inclusion criteria, the evidence was distributed across diverse neurotoxicant exposure categories, occupational populations, study designs, cognitive reserve operationalizations, neuroimaging methodologies, and brain aging outcomes. This heterogeneity limited direct comparison across studies and the feasibility of quantitative synthesis, making it difficult to determine the extent to which observed associations were comparable across occupational and exposure contexts. The broad grouping of brain aging outcomes also warrants acknowledgement, as cognitive decline, neuroimaging changes, neural damage biomarkers, dementia, and neurodegenerative mortality reflect different stages and manifestations of brain aging and are not directly comparable.

Fourth, the inclusion of one meta-analysis alongside primary studies also warrants consideration. Vlasak et al. (29) synthesized findings from 22 primary studies, and it is possible that some of those primary studies examined populations or exposures relevant to this review. The reference list of Vlasak et al. (29) was cross-checked against the 16 included studies, and no overlap was identified; however, the potential for residual overlap should be acknowledged as a limitation.

Fifth, the review was restricted to English-language publications and studies published from 2011 onward. Although the publication period was selected to capture the period following the development of contemporary cognitive reserve frameworks and the subsequent application of related approaches in occupational research, these eligibility restrictions may have resulted in the omission of relevant evidence published in other languages or before 2011. Additionally, a formal validation of the search strategy against a predefined set of key papers was not conducted, which may limit confidence in the comprehensiveness of the retrieved literature. Consequently, the findings may not fully represent the historical or international breadth of research on occupational neurotoxicant exposure, cognitive reserve, and brain aging.

Sixth, the review protocol was registered on the Open Science Framework during the conduct of the review rather than before its commencement, which may limit the value of the registration as a prospective safeguard against *post hoc* modification of the review process. The following methodological decisions were established before the literature search was conducted: the research questions, the PCC eligibility criteria, the database selection, the search term structure across three concept blocks, the two-stage screening process, and the data charting approach.

## 5 Conclusion

This scoping review mapped the available evidence linking occupational neurotoxicant exposure with cognitive reserve and brain aging outcomes in adult populations. Across the 16 included studies, diverse occupational neurotoxicant exposures were associated with a range of adverse cognitive and neurological findings, including cognitive impairment, structural and functional brain alterations, and biomarkers of neural injury. The findings were heterogeneous across exposure assessment methods, study designs, neuroimaging methodologies, cognitive reserve operationalizations, and brain aging outcomes. Nevertheless, the convergence of cognitive, neuroimaging, and neurological findings across diverse occupational neurotoxicant exposures suggests that occupational neurotoxicant exposure may contribute to processes relevant to brain aging, although the available evidence does not establish causality.

Cognitive reserve emerged as an important but inconsistently examined construct within the literature. Six of the 16 studies did not explicitly operationalize cognitive reserve, while the remaining studies used heterogeneous approaches to examine reserve or related characteristics, including educational attainment, biological susceptibility factors, and neuroimaging measures of brain structure or function. Importantly, the neuroimaging findings should not be interpreted as direct evidence of reduced or preserved cognitive reserve. Rather, they provide evidence of neural alterations that may help explain how occupational exposure is associated with cognitive outcomes and how individual differences in susceptibility may influence those outcomes. The available evidence therefore supports greater integration of cognitive reserve into occupational neurotoxicology research while emphasizing the need for clearer conceptual and methodological distinctions among cognitive reserve, brain structure, brain function, and biological susceptibility.

Important evidence gaps remain. The literature was characterized by a predominance of cross-sectional and case–control designs, limited evidence within individual neurotoxicant exposure categories, substantial heterogeneity in exposure and outcome assessment, and uneven geographical representation. Future research should prioritize prospective occupational cohorts incorporating quantitative and repeated exposure assessment, longitudinal cognitive evaluation, explicit multidimensional measures of cognitive reserve, and multimodal neuroimaging. Such studies could clarify the temporal sequence of exposure-related neurological changes, determine whether cognitive reserve modifies the cognitive expression of neurobiological changes, and examine factors that may account for variation in outcomes among similarly exposed workers. Greater representation of underrepresented occupational populations and geographical regions will also be important for determining the generalizability and context-dependence of observed associations.

From an occupational health perspective, the current evidence supports continued emphasis on primary prevention, exposure control, exposure monitoring, and appropriate occupational health surveillance rather than establishing differential occupational protection on the basis of cognitive reserve, educational attainment, or genetic characteristics. Continued efforts to identify and control neurotoxicant hazards, strengthen longitudinal occupational health surveillance, and build research capacity may help detect emerging neurological effects and inform more effective strategies for protecting workers’ long-term cognitive and brain health.

## Glossary

ACE: Angiotensin Converting Enzyme
APOE: Apolipoprotein E
DSST: Digit Symbol Substitution Test
DTI: Diffusion Tensor Imaging
fMRI: Functional Magnetic Resonance Imaging
ILO: International Labour Organization
JBI: Joanna Briggs Institute
LMICs: Low- and Middle-Income Countries
MCI: Mild Cognitive Impairment
MMSE: Mini-Mental State Examination
MoCA: Montreal Cognitive Assessment
MRI: Magnetic Resonance Imaging
NIOSH: National Institute for Occupational Safety and Health
OSF: Open Science Framework
PAH: Polycyclic Aromatic Hydrocarbon
PCC: Population–Concept–Context
PRISMA-ScR: Preferred Reporting Items for Systematic Reviews and Meta-Analyses Extension for Scoping Reviews
rs-fMRI: Resting-State Functional Magnetic Resonance Imaging
VDR: Vitamin D Receptor
WM: White Matter
