# Supplementary material for: Occupational neurotoxicant exposures, cognitive reserve, and brain aging: a scoping review
Source: Front Public Health. 2026 Sep 29;14:1971914. doi: 10.3389/fpubh.2026.1971914 (PMC13659289; doi:10.3389/fpubh.2026.1971914)
Supplement: Supplementary file 2 [file Supplementary_file_2.DOCX]

**Supplementary File 2. Full Electronic Search Strategies**

All searches were conducted in July 2026. Database-specific syntax modifications were applied where necessary.

**PubMed/MEDLINE**

( "Occupational Exposure"[MeSH Terms] OR occupational exposure*[tiab] OR workplace exposure*[tiab] OR work-related exposure*[tiab] OR occupational neurotoxicant*[tiab] OR occupational neurotoxic*[tiab] OR neurotoxicant exposure*[tiab] OR occupational chemical exposure*[tiab] OR "Lead"[MeSH Terms] OR lead exposure*[tiab] OR blood lead[tiab] OR "Mercury"[MeSH Terms] OR mercury exposure*[tiab] OR methylmercury[tiab] OR "Manganese"[MeSH Terms] OR manganese exposure*[tiab] OR "Arsenic"[MeSH Terms] OR arsenic exposure*[tiab] OR "Cadmium"[MeSH Terms] OR cadmium exposure*[tiab] OR "Solvents"[MeSH Terms] OR organic solvent*[tiab] OR solvent exposure*[tiab] OR "Pesticides"[MeSH Terms] OR pesticide exposure*[tiab] OR organophosphate*[tiab] OR pyrethroid*[tiab] OR welding fume*[tiab] OR exhaust fume*[tiab] OR polycyclic aromatic hydrocarbon*[tiab] OR "Metals, Heavy"[MeSH Terms] OR heavy metal*[tiab] ) AND ( "Cognitive Reserve"[MeSH Terms] OR cognitive reserve[tiab] OR brain reserve[tiab] OR neural reserve[tiab] OR neurocognitive reserve[tiab] OR cognitive resilience[tiab] OR brain resilience[tiab] OR brain maintenance[tiab] OR cognitive buffer*[tiab] OR "Educational Status"[MeSH Terms] OR educational attainment[tiab] OR premorbid IQ[tiab] OR premorbid intelligence[tiab] OR premorbid functioning[tiab] OR occupational complexity[tiab] OR reserve proxy[tiab] OR reserve proxies[tiab] OR brain age[tiab] OR brain age gap[tiab] OR biological brain age[tiab] OR functional connectivity[tiab] OR cortical thickness[tiab] OR white matter integrity[tiab] OR cognitive trajectory[tiab] OR neuroprotective[tiab] OR neural efficiency[tiab] OR protective factor*[tiab] OR cognitive protection[tiab] OR reserve moderator[tiab] OR effect modification[tiab] OR cognitive moderator[tiab] ) AND ( "Cognitive Aging"[MeSH Terms] OR cognitive aging[tiab] OR cognitive ageing[tiab] OR "Aging"[MeSH Terms] OR brain aging[tiab] OR brain ageing[tiab] OR "Cognitive Dysfunction"[MeSH Terms] OR cognitive impairment*[tiab] OR "Mild Cognitive Impairment"[MeSH Terms] OR mild cognitive impairment[tiab] OR MCI[tiab] OR "Dementia"[MeSH Terms] OR dementia[tiab] OR "Alzheimer Disease"[MeSH Terms] OR Alzheimer*[tiab] OR vascular dementia[tiab] OR cognitive decline[tiab] OR neurocognitive decline[tiab] OR "Neurodegenerative Diseases"[MeSH Terms] OR neurodegeneration[tiab] OR neurodegenerative[tiab] OR brain atrophy[tiab] OR cerebral atrophy[tiab] OR white matter hyperintensit*[tiab] OR cognitive function*[tiab] OR neuropsychological[tiab] OR neurobehavioral*[tiab] OR neurobehavioural*[tiab] OR cognitive performance[tiab] OR cognitive outcome*[tiab] ) Filters: English, Humans, from 2011 - 3000/12/12

Records retrieved: 410

**Scopus**

TITLE-ABS-KEY("occupational exposure" OR "workplace exposure" OR "work-related exposure" OR "occupational neurotoxicant*" OR "neurotoxicant exposure*" OR "lead exposure" OR "blood lead" OR "mercury exposure" OR "manganese exposure" OR "arsenic exposure" OR "cadmium exposure" OR "organic solvent*" OR "solvent exposure*" OR "pesticide exposure*" OR "organophosphate*" OR "welding fume*" OR "exhaust fume*" OR "heavy metal*") AND TITLE-ABS-KEY("cognitive reserve" OR "brain reserve" OR "neural reserve" OR "cognitive resilience" OR "brain resilience" OR "educational attainment" OR "premorbid IQ" OR "occupational complexity" OR "reserve proxy" OR "brain age gap" OR "functional connectivity" OR "cortical thickness" OR "white matter integrity" OR "neuroprotective" OR "protective factor*" OR "effect modification") AND TITLE-ABS-KEY("cognitive aging" OR "cognitive ageing" OR "brain aging" OR "brain ageing" OR "cognitive impairment*" OR "mild cognitive impairment" OR "dementia" OR "Alzheimer*" OR "cognitive decline" OR "neurodegeneration" OR "neurodegenerative" OR "brain atrophy" OR "cognitive function*" OR "neuropsychological" OR "neurobehavioral*" OR "neurobehavioural*") AND PUBYEAR > 2010 AND LANGUAGE(english) AND DOCTYPE(ar OR re)

Records retrieved: 323

**Embase**

('occupational exposure':ti,ab OR 'workplace exposure':ti,ab OR 'work-related exposure':ti,ab OR 'occupational neurotoxicant*':ti,ab OR 'neurotoxicant exposure*':ti,ab OR 'lead exposure':ti,ab OR 'blood lead':ti,ab OR 'mercury exposure':ti,ab OR 'manganese exposure':ti,ab OR 'arsenic exposure':ti,ab OR 'cadmium exposure':ti,ab OR 'organic solvent*':ti,ab OR 'solvent exposure*':ti,ab OR 'pesticide exposure*':ti,ab OR 'organophosphate*':ti,ab OR 'welding fume*':ti,ab OR 'exhaust fume*':ti,ab OR 'heavy metal*':ti,ab) AND ('cognitive reserve':ti,ab OR 'brain reserve':ti,ab OR 'neural reserve':ti,ab OR 'cognitive resilience':ti,ab OR 'brain resilience':ti,ab OR 'educational attainment':ti,ab OR 'premorbid iq':ti,ab OR 'occupational complexity':ti,ab OR 'reserve proxy':ti,ab OR 'brain age gap':ti,ab OR 'functional connectivity':ti,ab OR 'cortical thickness':ti,ab OR 'white matter integrity':ti,ab OR 'neuroprotective':ti,ab OR 'protective factor*':ti,ab OR 'effect modification':ti,ab) AND ('cognitive aging':ti,ab OR 'cognitive ageing':ti,ab OR 'brain aging':ti,ab OR 'brain ageing':ti,ab OR 'cognitive impairment*':ti,ab OR 'mild cognitive impairment':ti,ab OR 'dementia':ti,ab OR 'alzheimer*':ti,ab OR 'cognitive decline':ti,ab OR 'neurodegeneration':ti,ab OR 'neurodegenerative':ti,ab OR 'brain atrophy':ti,ab OR 'cognitive function*':ti,ab OR 'neuropsychological':ti,ab OR 'neurobehavioral*':ti,ab OR 'neurobehavioural*':ti,ab) AND 'human'/de AND [2011-2026]/py

Records retrieved: 111

**APA PsycInfo**

AB (occupational exposure OR workplace exposure OR work-related exposure OR occupational neurotoxicant* OR neurotoxicant exposure* OR lead exposure OR blood lead OR mercury exposure OR manganese exposure OR arsenic exposure OR cadmium exposure OR organic solvent* OR solvent exposure* OR pesticide exposure* OR organophosphate* OR welding fume* OR exhaust fume* OR heavy metal*) AND AB (cognitive reserve OR brain reserve OR neural reserve OR cognitive resilience OR brain resilience OR educational attainment OR premorbid IQ OR occupational complexity OR reserve proxy OR brain age gap OR functional connectivity OR cortical thickness OR white matter integrity OR neuroprotective OR protective factor* OR effect modification) AND AB (cognitive aging OR cognitive ageing OR brain aging OR brain ageing OR cognitive impairment* OR mild cognitive impairment OR dementia OR Alzheimer* OR cognitive decline OR neurodegeneration OR neurodegenerative OR brain atrophy OR cognitive function* OR neuropsychological OR neurobehavioral* OR neurobehavioural*)

Limiters applied via Search Options:

- Publication date: 2011–2026
- Language: English
- Field: Abstract (AB)

Records retrieved: 46
