# Supplementary material for: Occupational neurotoxicant exposures, cognitive reserve, and brain aging: a scoping review
Source: Front Public Health. 2026 Sep 29;14:1971914. doi: 10.3389/fpubh.2026.1971914 (PMC13659289; doi:10.3389/fpubh.2026.1971914)
Supplement: Supplementary file 3 [file Table_1.DOCX]

**Supplementary Table 1.** Characteristics of included studies (n = 16)

| **#** | **Author/Year** | **Country** | **Design** | **N** | **Population** | **Neurotoxicant** | **Exposure Measure** | **Cognitive Reserve Operationalization** | **Brain Aging Outcome** | **Key Finding** |
| --- | --- | --- | --- | --- | --- | --- | --- | --- | --- | --- |
| 1 | Zhao et al. 2023 | China | Case-control | 60 | Aluminum factory workers | Aluminum | Plasma aluminum concentration | Neuroimaging — DMN functional connectivity (fMRI) | Cognitive decline — MoCA | DMN1 functional connectivity partially mediated Al-MoCA relationship — mediation effect 25.80% |
| 2 | Vlasak et al. 2019 | Multiple | Meta-analysis | 3,849 (22 studies) | Adults with occupational or environmental lead exposure — occupational biomonitoring context | Lead | Blood lead concentration | None explicit | Cognitive and sensorimotor performance | Higher blood lead associated with poorer neurocognitive performance — Hedges g=0.09 per 10 μg/dL increase |
| 3 | Juárez-Pérez et al. 2019 | Mexico | Cross-sectional | 208 | Paint factory workers | Toluene, xylene, benzene | Personal air monitoring — cumulative toluene exposure index | None explicit | Cognitive and sensorimotor performance | Higher cumulative toluene exposure associated with poorer neuropsychological and sensorimotor performance across multiple domains |
| 4 | Kim et al. 2022 | South Korea | Longitudinal — 2-year | 301 | Firefighters — FRESH cohort | PAHs | Urinary PAH metabolites | Neuroimaging — cortical thickness and subcortical volume (MRI) | Cortical thickness reduction and subcortical volume change | PAH metabolites associated with cortical thickness reduction and subcortical volume change over 2-year follow-up; hypertension accelerated degenerative PAH effects |
| 5 | Wu et al. 2023 | China | Cross-sectional | 186 | Steel mill workers | Mixed metals — Ni, Co, Pb, As, Fe, Sr | Urinary metal concentrations — ICP-MS | None explicit | Neural damage biomarkers | Metal mixture exposure associated with neural damage biomarkers — Ni, Co, Pb, Sr, As and Fe showed adverse effects; Zn showed neuroprotective effects |
| 6 | James et al. 2011 | USA | Longitudinal — 5-year | 353 | Former organolead workers and controls | Lead — organolead | Bone lead — tibia | Genetic — APOE, ACE, VDR genotypes | Brain volume decline and white matter lesion progression (MRI) | APOE ε3/ε4 and ε4/ε4 genotypes associated with less WM volume decline; VDR FokI ff genotype associated with increased WM lesions |
| 7 | Meng et al. 2019 | China | Case-control | 853 | Aluminum factory workers on duty | Aluminum | Plasma aluminum concentration | Proxy-based — educational attainment as reserve moderator | Mild cognitive impairment | Higher plasma aluminum associated with increased MCI risk — AOR=2.24; higher education protective against aluminum-induced MCI — AOR=0.36 |
| 8 | Sabbath et al. 2012 | France | Prospective cohort | 4,134 | GAZEL cohort employees | Chlorinated solvents, petroleum solvents, benzene, nonbenzene aromatic solvents | Job-exposure matrix — lifetime cumulative | Proxy-based — educational attainment | Cognitive impairment — DSST | Solvent exposure was associated with cognitive impairment among less-educated workers, whereas this association was not observed among more-educated workers, suggesting a potential modifying role of education as a proxy for cognitive reserve. |
| 9 | Zhang et al. 2024a | China | Cross-sectional | 49 | Male aluminum-exposed workers — >10 years occupational exposure | Aluminum | Plasma aluminum concentration | Neuroimaging — gray/white matter functional networks (fMRI) | Cognitive impairment — MCI assessed by MoCA — executive control and limbic network disruption | Occupational aluminum exposure negatively correlated with MoCA scores — reduced FC in executive control and limbic networks resulting in MCI |
| 10 | Zhang et al. 2024b | China | Cross-sectional | 52 | Male aluminum-exposed workers — >10 years occupational exposure | Aluminum | Plasma aluminum concentration — ICP-MS | Neuroimaging — white matter integrity (DTI) + functional connectivity (rs-fMRI) | Mild cognitive impairment — MoCA — WM integrity and FC changes in fornix/stria terminalis | Plasma aluminum negatively correlated with MoCA scores — WM integrity and FC differences in right fornix/stria terminalis represent neural mechanisms of Al-induced MCI |
| 11 | Lee et al. 2024 | USA/South Korea | Cross-sectional | 73 | Adults with history of mixed metal exposure from welding fumes | Mixed metals — manganese and copper primary | Whole blood metal concentrations + quantitative brain MRI — exposure history | Neuroimaging — MTL DTI metrics — hippocampus, entorhinal, parahippocampal cortices | MTL structural changes and neuropsychological performance — processing speed, executive function, visuospatial, story recall | Mixed metal exposure associated with higher MTL diffusivity and poorer neuropsychological performance — blood Mn and Cu predicted entorhinal diffusivity and lower Delayed Story Recall; mediation through parahippocampal FA |
| 12 | Liang et al. 2025 | USA | Cross-sectional — NHANES | 1,110 | Older adults ≥60 years with occupational exhaust fume exposure history | Occupational exhaust fumes — PAHs and particulate matter | Self-reported occupational exhaust fume exposure history | None explicit | Cognitive decline — delayed memory, verbal fluency, processing speed | Occupational exhaust fume exposure associated with domain-specific cognitive decline — delayed memory OR=2.55, verbal fluency OR=2.41, processing speed OR=1.95; dose-response with 15–25 year latency period |
| 13 | Zbysinski et al. 2026 | USA | Retrospective cohort | 6,403 | Fernald uranium processing workers | Ionizing radiation + machining fluids | Cumulative brain radiation dose — mGy + job-exposure matrix | None explicit | Neurodegenerative mortality | Chemical co-exposures — particularly machining fluids — modified radiation-neurodegeneration relationship; null finding for radiation dose alone — HR=1.01 (95% CI 0.96–1.06) |
| 14 | Adani et al. 2020 | Italy | Case-control | 112 | Early-onset dementia patients and controls | Multiple chemical classes — aluminum, pesticides, dyes, paints, thinners | Self-reported occupational exposure questionnaire | None explicit | Early-onset dementia — AD and FTD | Occupational exposure to aluminum, pesticides, dyes, paints and thinners associated with increased FTD risk — chemical exposures not significantly associated with AD |
| 15 | Shi et al. 2025 | China | Cross-sectional | 49 | Smelting workers | Lead | Blood lead concentration | Neuroimaging — ReHo, DC, functional connectivity (rs-fMRI) | Cognitive deficit — MoCA and MMSE | Occupational lead exposure associated with insula functional connectivity disruption — negative correlations between blood lead and FC values; insula identified as pivotal region in lead-associated neurocognitive impairment |
| 16 | Reneman et al. 2016 | Netherlands | Case-control | 23 | Aircrew members with cognitive complaints | Organophosphates — engine oil fumes | Estimated flight hours | Neuroimaging — white matter microstructure (DTI) | Cognitive impairment — neuropsychological performance and reduced fMRI brain activation | Aircrew showed greater cognitive impairment and WM microstructure disruption vs controls — cognitive impairment extent strongly associated with WM integrity; flight hours not associated with cognitive impairment |

***Abbreviations:*** *ACE = angiotensin converting enzyme; AD = Alzheimer’s disease; AFT = Animal Fluency Test; APOE = apolipoprotein E; AOR = adjusted odds ratio; CERAD-WL = Consortium to Establish a Registry for Alzheimer’s Disease Word Learning subtest; DA = dopamine; DC = degree centrality; DMN = default mode network; DSST = Digit Symbol Substitution Test; DTI = diffusion tensor imaging; FA = fractional anisotropy; FC = functional connectivity; FRESH = Firefighters Research on Enhancement of Safety and Health; FTD = frontotemporal dementia; FX/ST = fornix/stria terminalis; GAZEL = GAZ et ELectricité (French national gas and electricity workers cohort); ICP-MS = inductively coupled plasma mass spectrometry; K-XRF = K-shell X-ray fluorescence; MCI = mild cognitive impairment; mGy = milligray; MMSE = Mini-Mental State Examination; MoCA = Montreal Cognitive Assessment; MRI = magnetic resonance imaging; MTL = medial temporal lobe; NfL = neurofilament light chain; NHANES = National Health and Nutrition Examination Survey; PAH = polycyclic aromatic hydrocarbon; PD = Parkinson’s disease; PM2.5 = fine particulate matter; PRL = prolactin; ReHo = regional homogeneity; rs-fMRI = resting-state functional magnetic resonance imaging; S1P = sphingosine-1-phosphate; VDR = vitamin D receptor; VOC = volatile organic compound; WM = white matter; WML = white matter lesions.*
